# Mass Cytometry Studies of Patients With Autoimmune Endocrine Diseases Reveal Distinct Disease-Specific Alterations in Immune Cell Subsets

**DOI:** 10.3389/fimmu.2020.00288

**Published:** 2020-02-21

**Authors:** Louise Magnusson, Hugo Barcenilla, Mikael Pihl, Sophie Bensing, Daniel Espes, Per-Ola Carlsson, Rosaura Casas

**Affiliations:** ^1^Department of Medical Sciences, Uppsala University, Uppsala, Sweden; ^2^Division of Children and Women Health, Department of Biomedical and Clinical Sciences, Linköping University, Linköping, Sweden; ^3^Core Facility Flow Cytometry Unit, Faculty of Medicine, Linköping University, Linköping, Sweden; ^4^Department of Molecular Medicine and Surgery, Karolinska Institutet, Stockholm, Sweden; ^5^Department of Medical Cell Biology, Uppsala University, Uppsala, Sweden

**Keywords:** mass cytometry (CyTOF), type 1 diabetes, Hashimoto's thyroiditis, Graves' disease, Addison's disease, antigen-presenting cells, NK cells, T cells

## Abstract

Although there is evidence that autoimmune diseases share similar immunogenetic mechanisms, studies comparing peripheral CD45^+^ cells from patients with autoimmune endocrine diseases in parallel are limited. In this study, we applied high-dimensional single-cell mass cytometry to phenotypically characterize PBMC from patients with new-onset (N-T1D) and long-standing type 1 diabetes, Hashimoto's thyroiditis (HT), Graves' disease and autoimmune Addison's disease (AD), as well as healthy controls. The frequency of CD20^lo^CD27^hi^CD38^hi^HLA-DR^int^ plasmablasts, CD86^+^CD14^lo^CD16^+^ non-classical monocytes and two subsets of CD56^dim^HLA-DR^+^IFN-γ^+^ NK cells were increased in patients with HT. Subsets of CD56^dim^CD69^+^HLA-DR^−^ NK cells and CD8^+^ TEMRA cells, both expressing IFN-γ, were expanded and reduced, respectively, in the N-T1D group. In addition, patients with AD were characterized by an increased percentage of central memory CD8^+^ T cells that expressed CCR4, GATA3, and IL-2. We demonstrate that patients with N-T1D, HT, and AD had altered frequencies of distinct subsets within antigen-presenting and cytotoxic cell lineages. Previously unreported alterations of specific cell subsets were identified in samples from patients with HT and AD. Our study might contribute to a better understanding of shared and diverging immunological features between autoimmune endocrine diseases.

## Introduction

Despite that targets and symptoms vary between autoimmune diseases, several features underlying autoimmunity are considered to be shared. This idea is not novel as “mosaic of autoimmunity” and “autoimmune tautology” are established concepts (1, 2). These concepts declare that genetic and epigenetic factors predispose individuals to fail central and peripheral tolerance mechanisms. An autoimmune response can later be triggered by inauspicious hormonal background and environmental factors, leading to overt disease. This model pertains all autoimmune diseases, although specific genetic, hormonal, immunological, and environmental factors and their interactions determine the final disease.

As a large proportion of patients with type 1 diabetes (T1D), Hashimoto's thyroiditis (HT), Graves' disease (GD), and autoimmune Addison's disease (AD) develop poly-autoimmunity or Autoimmune Polyendocrine Syndrome (2–5), it can be expected that patients with these diseases have similar immunological deviations. Autoantibodies produced by B cells are the most evident immunological feature in patients with T1D, HT, GD and AD, which can directly cause symptoms and modulate the function of other immune cells (6–8). Evidence suggests that monocytes (Mo) and antigen-presenting cells are also involved in the inflammatory process by the secretion of cytokines that induce differentiation of pro-inflammatory effector T cells (6, 9). Indeed, a typical feature for T1D, HT, GD, and AD is the presence of CD8^+^ and CD4^+^ T cells that lyse target cells and secrete IFN-γ and TNF-α upon autoantigen stimulation (6, 10–13). Studies addressing T cells in autoimmune endocrine diseases have nevertheless described a more complex process, as increased Th2-/Th17-biased immune responses (13–15) and a defective immunomodulation by Tregs (16, 17) have been reported. An issue with the abovementioned studies is that no or few simultaneous comparisons between diseases have been made, as patients with only one autoimmune condition have been mostly recruited. Another issue is that immune deviations associated with autoimmune endocrine diseases have been examined within a limited number of major cell lineages due to methodological limitations.

Mass cytometry is a high-dimensional single-cell technology that enables simultaneous characterization of the immune system, which has yielded novel information about T1D (18–20), systemic lupus erythematous (21) and rheumatoid arthritis (22). A limited number of studies have used this approach to compare different diseases in parallel (23, 24), but none have focused on immunological features in patients with autoimmune endocrine diseases. In our study, we used mass cytometry to perform a deep characterization and simultaneous comparison of PBMC from patients with T1D, HT, GD, and AD. By applying hierarchical dimensionality reduction and clustering analyses, we identified distinct subsets within B cells, Mo, NK cells and memory CD8^+^ T cells that were altered in patients with new-onset T1D (N-T1D), HT, and AD.

## Results

We used a mass cytometry panel with 32 lanthanide-labeled antibodies, including markers for lineage, activation/function, differentiation, cytokines, chemokine receptors and transcription factors, to phenotypically characterize PBMC. Samples from patients with N-T1D, long-standing T1D (L-T1D), HT, GD, and AD, as well as healthy controls (HC), were examined in this study. The hierarchical stochastic neighbor embedding (HSNE) dimensionality reduction analysis and unsupervised Gaussian mean-shift (GMS) clustering were applied to reveal the composition of single live CD45^+^ cells (Supplementary Figure 1A).

**Figure 1:**
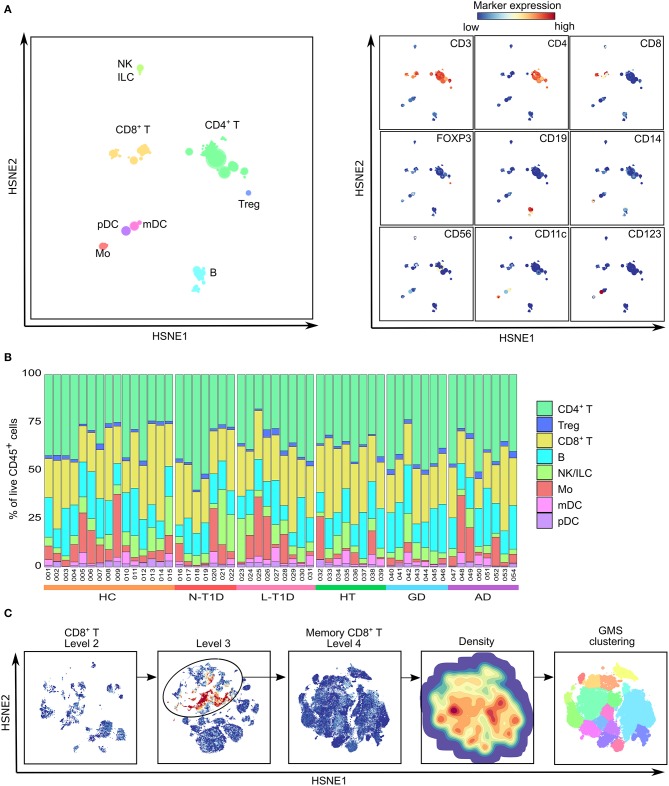
Delineation of major cell lineages within PBMC. **(A)** A HSNE-embedding at the overview level showing a collective analysis of PBMC from healthy controls (HC, *n* = 15) and patients with new-onset (N-T1D, *n* = 7) and long-standing (L-T1D, *n* = 9) type 1 diabetes, Hashimoto's thyroiditis (HT, *n* = 8), Graves' disease (GD, *n* = 7) and autoimmune Addison's disease (AD, *n* = 8). Eight major cell lineages (left) were defined based on the ArcSinh5-transformed expression of conventional lineage markers (right). **(B)** Proportions (%) of the different lineages in live CD45^+^ cells. Numbers and colors below the bar graph represent individuals (001-054) and groups, respectively. **(C)** Example of hierarchal data exploration applied on all cell lineages. The CD8^+^ T cell lineage from the overview level was selected and embedded at more detailed levels. Memory CD8^+^ T cells (black circle) were selected at level 3 based on CD45RO expression. At level 4, unsupervised Gaussian mean-shift (GMS) clustering used the local probability density of HSNE-embedded cells (density map) to identify phenotypically distinct clusters (color partitions). ILC, innate lymphoid cell; HSNE, hierarchical stochastic neighbor embedding.

Based on the expression of lineage markers, eight major cell lineages were identified: CD19^+^ B cells, CD14^+^ Mo, lin^−^CD56^+^ NK cells and innate lymphoid cells (ILC), CD3^+^CD8^+^ and CD3^+^CD4^+^ T cells, CD4^+^CD25^hi^CD127^lo^FOXP3^+^ Tregs, lin^−^CD11c^+^CD123^−^ myeloid dendritic cells (mDC) and lin^−^CD11c^−^CD123^+^ plasmacytoid dendritic cells (pDC) (Figure 1A). Quantification of their relative frequencies in each sample corroborated that CD3^+^ T cells were the most abundant cell type within CD45^+^ cells, whereas proportions of CD19^+^ B cells, CD14^+^ Mo and lin^−^CD56^+^ NK cells/ILC varied between subjects (Figure 1B). Despite the large individual variability, there were no differences in the frequency of major cell lineages between the groups. Every cell lineage was then selected and explored in detail by HSNE to identify clusters, as exemplified by CD8^+^ T cells (Figure 1C). Our results revealed a total of 125 phenotypically distinct subsets, illustrating a high degree of heterogeneity within PBMC (Supplementary Figure 1B). HSNE maps generated by the analyses of CD45^+^ cells were similar in patients and HC (Supplementary Figure 2). However, the statistical analysis of each cluster revealed alterations within some subsets of B cells, Mo, NK cells, and memory CD8^+^ T cells in three groups of patients.

### CD19^+^ B Cell and CD14^+^ Monocyte Subsets Were Specifically Altered in Patients With HT

**Figure 2:**
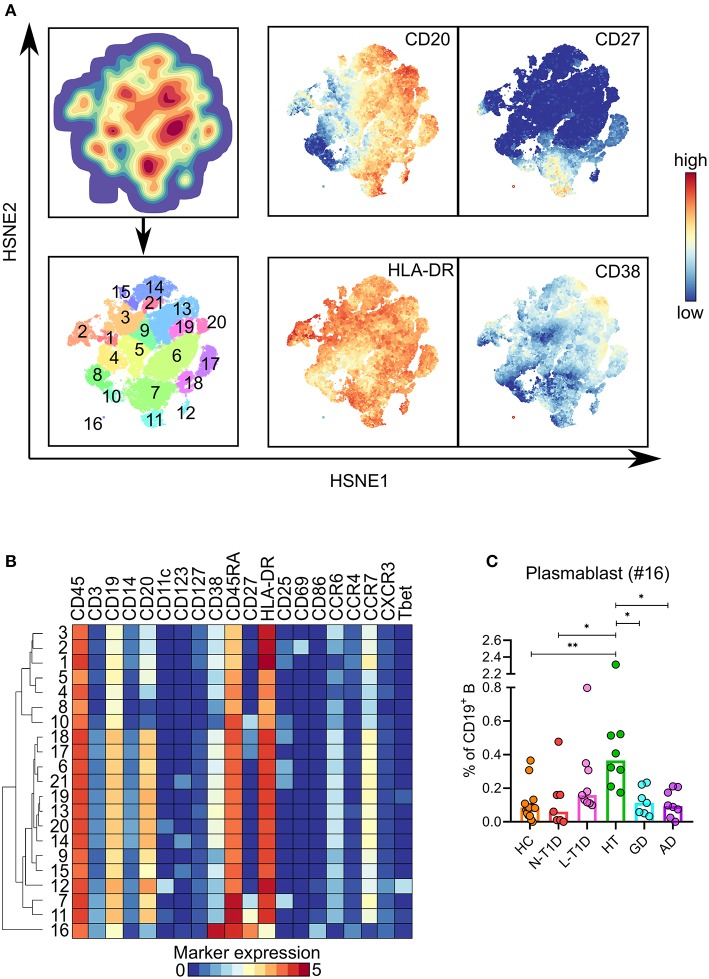
Expansion of plasmablasts in patients with Hashimoto's thyroiditis. **(A)** HSNE-embedding depicting the local probability density of CD19^+^ B cells (upper first left) and the subsets defined by Gaussian mean-shift clustering (lower first left). Relative expression of selected markers in embedded CD19^+^ B cells. **(B)** Heatmap showing ArcSinh5-transformed median expression of relevant markers within the clusters identified in (A). The dendrogram depicts hierarchical clustering of subsets. **(C)** Frequency of plasmablasts (#16) in healthy controls (HC, *n* = 15) and patients with new-onset (N-T1D, *n* = 7) and long-standing (L-T1D, *n* = 9) type 1 diabetes, Hashimoto's thyroiditis (HT, *n* = 8), Graves' disease (GD, *n* = 7) and autoimmune Addison's disease (AD, *n* = 8). Dots represent individual samples and bars indicate median. One-way ANOVA with Tukey's test for multiple comparisons, *p < 0.05 and **p < 0.01. HSNE, hierarchical stochastic neighbor embedding.

Clustering of the CD19^+^ B cell lineage defined 21 phenotypically distinct clusters, where the majority comprised CD27^−^ B cells (Figures 2A,B). Although CD20^lo^CD27^hi^CD38^hi^HLA-DR^int^ plasmablasts (B #16) only constituted 0.1–2% of CD19^+^ B cells, this cell subset was more frequent in patients with HT than in HC (*p* = 0.006) and patients with N-T1D (*p* = 0.04), GD (*p* = 0.04), and AD (*p* = 0.02, Figure 2C). We identified a cluster of CD11c^+^CD27^−^CXCR3^+^T-bet^+^ B cells (B #12), which have been previously associated with autoimmune diseases (25, 26). This cell subset was more frequent in patients with HT although it only reached a statistical difference with the N-T1D group (Supplementary Figure 3). The level of cytokine expression within the whole B cell lineage was low, which precluded us to define cytokine expression within specific clusters.

**Figure 3:**
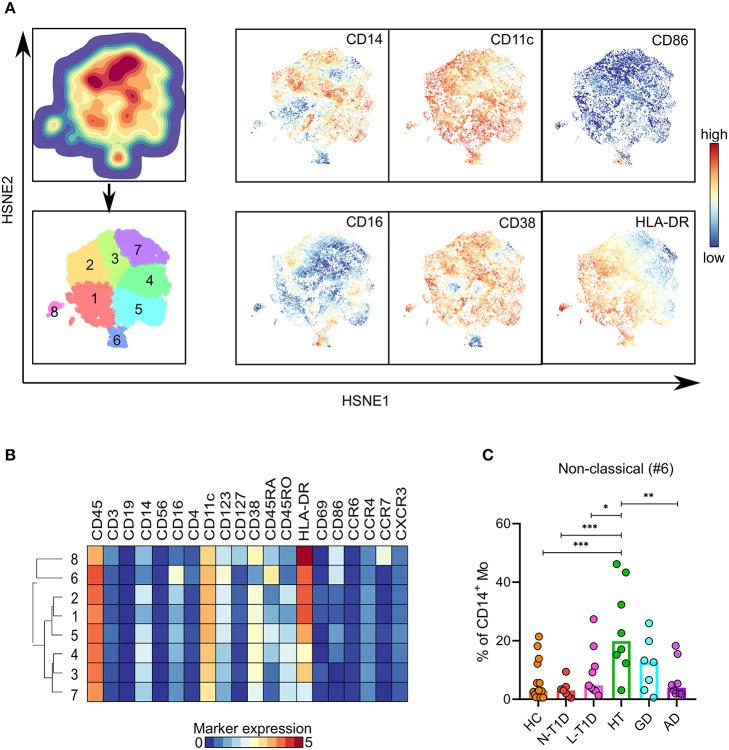
Higher frequency of non-classical monocytes in patients with Hashimoto's thyroiditis. **(A)** HSNE-embedding depicting the local probability density of CD14^+^ Mo (upper first left) and subsets defined by Gaussian mean-shift clustering (lower first left). Relative expression of selected markers in embedded CD14^+^ Mo. **(B)** Heatmap showing ArcSinh5-transformed median expression of relevant markers within the clusters identified in (A). The dendrogram depicts hierarchical clustering of subsets. **(C)** Frequency of non-classical Mo (#6) in healthy controls (HC, *n* = 15) and patients with new-onset (N-T1D, *n* = 7) and long-standing (L-T1D, *n* = 9) type 1 diabetes, Hashimoto's thyroiditis (HT, *n* = 8), Graves' disease (GD, *n* = 7) and autoimmune Addison's disease (AD, *n* = 8). Dots represent individual samples and bars indicate median. One-way ANOVA with Tukey's test for multiple comparisons, *p < 0.05, **p < 0.01, and ***p < 0.001. HSNE, hierarchical stochastic neighbor embedding; Mo, monocytes.

The hierarchical analysis of the CD14^+^ Mo lineage identified eight clusters (Figures 3A,B). Patients with HT had a higher frequency of CD86^+^CD14^lo^CD16^+^ non-classical Mo (#6) than HC (*p* = 0.0005) and patients with N-T1D (*p* = 0.0005), L-T1D (*p* = 0.01) and AD (*p* = 0.003, Figure 3C). In spite of evident disease-associated signatures in the lineage-context (Supplementary Figure 2), no other differences were observed between the groups. We were not able to detect cytokine expression in CD14^+^ Mo.

HSNE analysis of the mDC and pDC lineages identified six and four clusters, respectively (Supplementary Figures 4E,F). Neither cluster differed between the groups.

### Alterations in CD56^dim^ NK Cell Subsets Expressing IFN-γ Were Characteristic for Patients With N-T1D and HT

**Figure 4:**
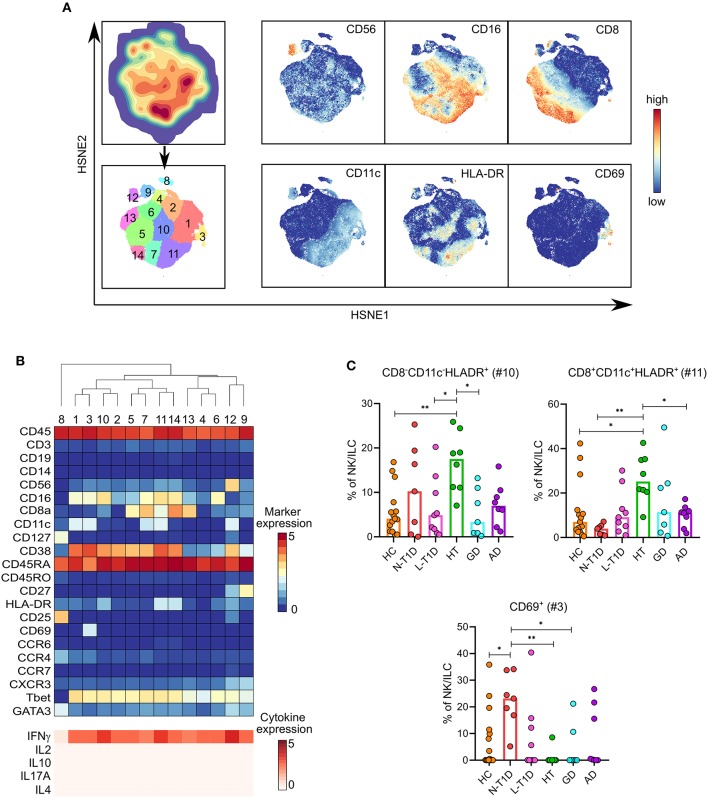
Alterations in distinct CD56^dim^ NK cell clusters in patients with new-onset type 1 diabetes and Hashimoto's thyroiditis. **(A)** HSNE-embedding depicting the local probability density of CD56^+^ NK cells and ILC (upper first left) and the subsets defined by Gaussian mean-shift clustering (lower first left). Relative expression of selected markers in embedded CD56^+^ NK cells and ILC. **(B)** Heatmap showing ArcSinh5-transformed median expression of relevant markers (top) and differential median ArcSinh5-expression of cytokines after PMA/ionomycin stimulation (bottom) within the clusters identified in (A). The dendrogram depicts hierarchical clustering of subsets. **(C)** Frequencies of CD69^+^ (#3), CD8^−^CD11c^−^HLA-DR^+^ (#10), and CD8^+^CD11c^+^HLA-DR^+^ (#11) NK cells in healthy controls (HC, *n* = 15) and patients with new-onset (N-T1D, *n* = 7) and long-standing (L-T1D, *n* = 9) type 1 diabetes, Hashimoto's thyroiditis (HT, *n* = 8), Graves' disease (GD, *n* = 7) and autoimmune Addison's disease (AD, *n* = 8). Dots represent individual samples and bars indicate median. One-way ANOVA with Tukey's test for multiple comparisons, *p < 0.05 and **p < 0.01. HSNE, hierarchical stochastic neighbor embedding; ILC, innate lymphoid cells.

Clustering of lin^−^CD56^+^ NK cells and ILC defined 14 subsets (Figure 4A). Thirteen of them expressed T-bet and IFN-γ, which could thus be classified as group 1 ILC (Figure 4B). A cluster of CD56^−^GATA3^+^ ILC2 (NK #8) also clustered within the NK cell lineage. Independent statistical analysis revealed that patients with N-T1D had a higher frequency of CD8^−^CD11c^+^CD56^dim^CD69^+^HLA-DR^−^IFN-γ^+^ NK cells (NK #3) than HC (*p* = 0.03) and patients with HT (*p* = 0.003) and GD (*p* = 0.03, Figure 4C). Two subsets of CD56^dim^HLA-DR^+^ NK cells were specifically abundant in patients with HT. The CD8^−^CD11c^−^ cluster (NK #10) was more frequent compared with HC (*p* = 0.004) and the L-T1D (*p* = 0.02) and GD groups (*p* = 0.02), whereas the frequency of CD8^+^CD11c^+^ cells (NK #11) was higher than in HC (*p* = 0.04) and the N-T1D (*p* = 0.003) and AD groups (*p* = 0.046). These two subsets expressed high levels of IFN-γ, which was comparable to the production by CD56^hi^ NK cells (NK #12) (Figure 4B).

### Distinct Memory Subsets and Altered Cytokine Expression in CD8^+^ T Cells Were Associated With Patients With N-T1D and AD

**Figure 5:**
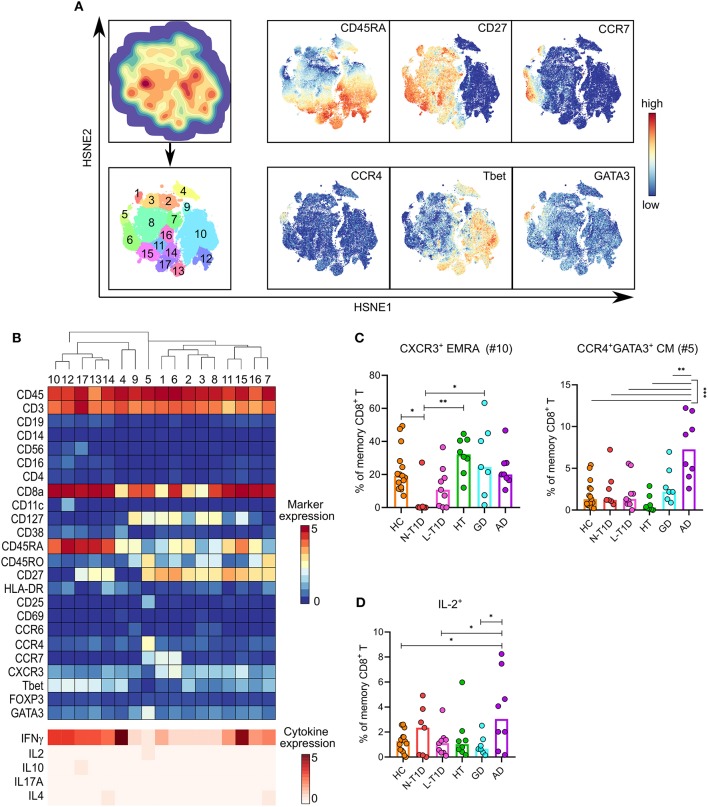
Alterations in distinct memory CD8^+^ T cell subsets with differential cytokine expression in patients with new-onset type 1 diabetes and Addison's disease. **(A)** HSNE-embedding depicting the local probability density of memory CD8^+^ T cells (upper first left) and the subsets defined by Gaussian mean-shift clustering (lower first left). Relative ArcSinh5-transformed expression of selected markers in HSNE-embedded memory CD8^+^ T cells. **(B)** Heatmap showing ArcSinh5-transformed median expression relevant markers (top) and differential median ArcSinh5-expression of cytokines after PMA/ionomycin stimulation (bottom) within the clusters identified in (A). The dendrogram depicts hierarchical clustering of subsets. **(C)** Frequencies of CXCR3^+^ EMRA (#10) and CCR4^+^GATA3^+^ CM (#5) T cells in healthy controls (HC, *n* = 15) and patients with new-onset (N-T1D, *n* = 7) and long-standing (L-T1D, *n* = 9) type 1 diabetes, Hashimoto's thyroiditis (HT, *n* = 8), Graves' disease (GD, *n* = 7) and autoimmune Addison's disease (AD, *n* = 8). **(D)** Frequency of memory CD8^+^ T cells expressing IL-2 after PMA/ionomycin stimulation. Dots represent individual samples and bars indicate median. One-way ANOVA with Tukey's test for multiple comparisons, *p < 0.05, **p < 0.01, and ***p < 0.001. CM, central memory; EMRA, CD45RA^+^ effector memory; HSNE, hierarchical stochastic neighbor embedding.

Seventeen memory CD8^+^ T cell subsets were identified by clustering analysis (Figures 5A,B). Patients with N-T1D had a lower frequency of CD45RA^+^CD27^−^CCR7^−^CXCR3^+^T-bet^+^IFN-γ^+^ TEMRA cells (CD8 #10) than HC (*p* = 0.04) and the HT (*p* = 0.003) and GD groups (*p* = 0.01, Figure 5C). A cluster of CCR4^+^GATA3^+^ central memory cells (CD8 #5) expressed low levels of IL-2, which was more frequent in patients with AD than in all other groups (*p* < 0.0001 vs. HC and HT, *p* = 0.0009 vs. N-T1D, *p* = 0.0001 vs. L-T1D, *p* = 0.004 vs. GD). Although the expression of IL-2 was low in CD8 #5, this subset accounted for most of the IL-2 expression within memory CD8^+^ T cells. The percentage of memory CD8^+^ T cells expressing IL-2 was also higher in the AD group than in HC (*p* = 0.02) and the L-T1D (*p* = 0.04) and GD groups (*p* = 0.045, Figure 5D).

Naïve and memory CD4^+^ T cells comprised 13 and 17 clusters, respectively, whereas 10 cell subsets were identified within the Treg lineage (Supplementary Figures 4A–C). Clustering of naïve CD8^+^ T cells identified 15 subsets (Supplementary Figure 4D). No differences in the frequency of these clusters were observed between the groups.

## Discussion

In this study, we took advantage of high-dimensional mass cytometry to phenotypically characterize PBMC from patients with T1D, HT, GD, and AD. We identified changes in plasmablasts, non-classical Mo and HLA-DR^+^ NK cells in patients with HT, whereas the N-T1D group had alterations in distinct NK and effector CD8^+^ T cell subsets. Moreover, patients with AD were characterized by an expansion of CCR4^+^GATA3^+^ central memory CD8^+^ T cells. As it has been hypothesized that these autoimmune endocrine diseases are chiefly driven by Th1- and cytotoxic immune responses (6, 10–13), it was expected that patients with established autoimmune endocrine diseases would have shared several immunological features. The majority of cell subsets were indeed similar between the patient groups, as well as in comparison with HC, but we were however able to identify distinct alterations in the N-T1D, HT, and AD groups.

A higher frequency of plasmablasts detected in patients with HT suggests that B cells are continuously activated after diagnosis and treatment. B cells might play an important role in HT, as an expansion of peripheral CD27^+^CD138^+^ plasma cells has been observed in patients with recent-onset of the disease (27). A role for B cells is further supported by a study demonstrating that intra-thyroidal germinal centers were common in patients with HT (28). It has been suggested that plasmablasts modulate effector functions of CD45^+^ cells via IgG/Fc-receptor signaling during HT (8, 29, 30). Peripheral expansion of plasmablasts in an autoimmune context has been attributed to CD11c^+^T-bet^+^ B cells (25, 26, 31), which are antigen-experienced CD27^lo^IgD^lo^ B cells that can differentiate into plasmablasts and produce autoantibodies more efficiently than CD27^+^ memory B cells (26). We observed that a subset of CD11c^+^CD27^−^CXCR3^+^T-bet^+^ B cells was more frequent in patients with HT, supporting the idea that these cells might promote a plasmablast expansion. Alterations within CD19^+^ B cell subsets were only found in patients with HT despite that autoantibodies are also present in T1D, GD and AD. Previous studies addressing peripheral B cells in T1D and GD have reported that frequencies were similar compared to HC (32–34) or that functional responses toward autoantigens were changed (35, 36).

Patients with HT were also characterized by an increased frequency of CD86^+^ non-classical Mo, which has been previously reported in patients with other autoimmune diseases including multiple sclerosis (37), systemic lupus erythematous (38), and primary biliary cirrhosis (39). Variations in sample material, separation protocols, detection methodology and definition of Mo subsets might explain why we did not detect a parallel reduction of classical Mo as observed in other studies (38, 39). A higher frequency of non-classical Mo might have a relevant role during HT, as this subset express markers related to antigen-presentation and secrete IL-1β/TNF-α to a higher degree than classical Mo (38). A parallel expansion of plasmablasts and CD86^+^ non-classical Mo in the HT group is an interesting observation, as anti-thyroid peroxidase antibodies have been shown to activate CD14^+^ Mo and thereby induce antibody-dependent cell-mediated cytotoxicity and cytokine secretion (8, 29, 30). Consistent with our results, studies on peripheral and tissue-resident CD14^+^ Mo from patients with T1D and GD have shown that the frequency of Mo was similar compared to HC (9, 40–42). Abovementioned studies revealed nevertheless that the secretion of pro-inflammatory cytokines was enhanced, implying that an altered function of CD14^+^ Mo is instead the mechanism during T1D and GD.

An expansion of two CD56^dim^ NK subsets, expressing HLA-DR at a higher intensity than CD56^hi^ NK cells, was also detected in samples from patients with HT. This is an interesting finding, as it has been described that CD56^hi^ NK cells normally express HLA-DR to a higher degree than CD56^dim^ NK subsets (43, 44). Intriguingly, it has been shown that CD56^dim^HLA-DR^+^ NK cells develop during pro-inflammatory conditions (45) and have better stimuli-induced secretion of IFN-γ than their HLA-DR^−^ counterparts (44, 45). Another function attributed to CD56^dim^HLA-DR^+^ NK cells is their capacity to induce differentiation of naïve CD4^+^ T cells into pre-central memory T cells (45). In our study, a high expression of IFN-γ in both CD56^dim^HLA-DR^+^ subsets might suggest that these cells can be involved in the maintenance of inflammatory immune responses in patients with HT. Several studies have reported an impaired cytotoxicity, yet a normal frequency, of peripheral CD3^−^CD56^+^ NK cells in adults with HT, GD, and AD (46–50). An impaired NK cell function has however been attributed to hormonal imbalances and treatment-induced immunosuppression (46–48, 50, 51).

Most studies addressing NK cells in T1D have focused on their cytotoxic function, whereas studies investigating the phenotype of cell subsets are limited (52, 53). It has been recently shown that children with newly diagnosed T1D had reduced frequencies of CD16^+^CD56^dim^ and CD16^−^CD56^dim^ NK cell subsets than HC (32). We have previously reported that multiple autoantibody-positive children, who later progressed to T1D, had higher frequencies of two CD8^+^CD16^+^CD56^dim^ NK cell subsets than autoantibody-negative individuals (18). By using the same mass cytometry panel, we found an expansion of activated mature/effector CD8^−^CD11c^+^CD16^+^CD56^dim^CD69^+^HLA-DR^−^ NK cells that expressed IFN-γ in adults with N-T1D. Expression of CD11c and CD69 on this peripheral cell subset is very intriguing, as these two markers are normally detected to a higher degree in tissue-resident NK cells (54, 55). In addition, it has been shown that CD69^+^ NK cells expressed IFN-γ to a higher degree than CD69^−^ NK cells (56). A higher frequency of activated NK cells with a pro-inflammatory phenotype in adults with newly diagnosed T1D might reflect the ongoing inflammatory process, as this alteration was not observed in samples from patients with L-T1D.

It was interesting that the N-T1D group had a lower frequency of CXCR3^+^T-bet^+^IFN-γ^+^ TEMRA cells within the CD8^+^ T cell lineage compared with HC. CD8^+^ TEMRA cells are terminally differentiated cells known to be highly cytotoxic due to their abundant perforin content, cytokine secretion and low activation threshold (57). Studies on pancreatic biopsies from patients with N-T1D have shown that CD8^+^ T cells are recruited to insulin-positive islets and are the most prevalent cell type during insulitis (10, 20, 58). Thus, a reduction of TEMRA cells in peripheral blood from newly diagnosed T1D patients might be explained by their migration into the pancreas, where they can contribute to β-cell apoptosis and dysfunction.

Studies addressing immune responses in patients with AD are scarce, as this is a rare condition that is often accompanied by other autoimmune endocrine diseases (59). Autoantibodies and chemokines in serum have been extensively studied (4, 60–62), whereas few studies have focused on cellular phenotype and function. It has been shown that CD8^+^ and CD4^+^ T cells from patients with AD proliferate and secrete IFN-γ upon stimulation with 21-hydroxylase (6, 11, 63). In this study, we found that CCR4^+^GATA3^+^ central memory CD8^+^ T cells were more frequent in patients with AD. This cell subset expressed IL-2 at low levels, which could be related to the higher frequency of memory CD8^+^ T cells expressing IL-2 in patients with AD. Our results further revealed that these “type 2” memory T cells also expressed CD25, but not FOXP3 or the early (CD69) and chronic (HLA-DR) activation markers. A similar central memory CD8^+^CD25^+^ T cell subset has been previously described, which had a Th2-like phenotype, expressed IL-2 and lacked regulatory functions (64, 65). Studies addressing corticosteroid treatment and CD8^+^ T cells have reported that the whole CD8^+^ T cell population is suppressed and/or depleted (66–68). As a deeper phenotypic characterization of CD8^+^ T cells was not made in previous studies, it cannot be excluded that an increase of a distinct Th2-like central memory CD8^+^ T cell subset in patients with AD might be the result of corticosteroid treatment. For instance, synthetic glucocorticoids selectively inhibited IFN-γ and IRF-1 expression in T cells, without affecting IL-4-signaling (69), leading to a shift toward Th2-biased immune responses (70, 71). Thus, an expansion of peripheral CCR4^+^GATA3^+^ central memory CD8^+^ T cells in patients with AD might be due to a drug-induced CD8^+^ T cell differentiation that does not reflect the pro-inflammatory environment in adrenal glands.

By using a high-dimensional single-cell technology, we were able to simultaneously study lineage, differentiation, activation, and functional markers in major immune cell populations. It could be argued that a limitation of the study is the size of the groups. To avoid methodological variations, intrinsic for this approach, it was important to limit the time window for sample analysis. As only patients diagnosed with one autoimmune endocrine disease were included, it was also difficult to recruit a large number of well-defined patients within a short time period. We were aware of a possible age variation between the groups, especially regarding patients with AD. However, isolated AD is not highly prevalent in Sweden (Swedish Addison Registry) and younger individuals with AD tend to develop poly-autoimmunity more frequently than older subjects (2, 5, 72). Although peripheral blood is not optimal to study immune responses in affected organs, biopsies from the pancreas and adrenal glands are rarely taken for medical and ethical reasons due to the severe complications entailing this procedure. To avoid immunological changes provoked by metabolic disturbances (51, 73), only individuals with good hormonal and metabolic control at the time of sampling were included in the study. All patients were medicated according to clinical standards and were treatment-responsive. As the treatments were aimed to normalize derangements in glucose control (T1D), thyroid status (HT and GD), or cortisol levels and electrolytes (AD), medicine doses might have varied between individuals within each disease group. One striking finding was that patients with HT had alterations within different cell lineages not observed in the other groups, especially when compared with the GD patients. Both HT and GD are autoimmune thyroid diseases, but their mechanisms involve different arms of the immune system, leading to distinct destructive effects on the thyroid gland (74). It has been suggested that HT is primarily caused by dysregulation of immune cells, as the disease is clustered with additional autoimmunity, especially against adrenal glands and β-cells, to a higher degree than GD (75). The immunological alterations in patients with established and treated HT might raise the question whether drugs targeting the immune system could be an attractive complement to conventional hormone replacement.

In conclusion, we show that patients with N-T1D, HT, and AD had altered frequencies of distinct clusters within antigen-presenting and cytotoxic cell lineages. Importantly, we were able to identify previously unreported alterations in rare cell subsets from patients with HT and AD. To our knowledge, there are no previous studies addressing differences in PBMC from patients with autoimmune endocrine diseases by a high-dimensional single-cell technology. Our results might contribute to a better understanding of shared and diverging immunological features between autoimmune endocrine diseases. This study was not designed to address the possible functional role of the identified clusters in the pathogenesis of the diseases, which should be addressed in future studies.

## Materials and Methods

### Study Design and Participants

Patients with N-T1D, L-T1D, HT, GD, or AD, as well as HC, were recruited at Uppsala University Hospital between January 2017 and June 2018. All subjects were 18–50 years old and had no signs of infections, malignancies or other diseases at time of inclusion. Patients had only one known autoimmune endocrine disease and did not present derangements in hormone production and autoantibodies for any of the other studied diseases. HC had no first-degree relatives with autoimmune endocrine diseases, no derangements in hormonal function and were negative for the tested disease-specific autoantibodies. All patients were actively treated for their respective diseases: intensive insulin therapy for T1D, levothyroxine for HT, block-replace therapy (thiamazole with addition of levothyroxine after 4 weeks) for GD and hydrocortisone for AD. Patients with GD had not been previously treated with radioiodine or ablative surgery.

The following parameters were measured at the Clinical Chemistry and Pharmacology Laboratory, Uppsala University Hospital, Sweden: creatinine (μmol/L), fasting glucose (mmol/L), glycated hemoglobin (mmol/mol), C-peptide (nmol/L), thyroid-stimulating hormone (mIE/L), free thyroxine (pmol/L), free triiodothyronine (pmol/L), serum cortisol (nmol/L), anti-glutamic acid decarboxylase (IE/mL), anti-tyrosine phosphatase like protein islet antigen-2 (kE/L), anti-thyroid peroxidase (kIE/L), thyroid-stimulating immunoglobulins (E/L) and anti-21-hydroxylase (kE/L). Characteristics of the groups are summarized in Table 1. Sex distribution and age were similar between the groups except that the N-T1D group was younger than the AD group (*p* < 0.05).

**Table 1:** Descriptive data of cohort groups.

**Parameter**	**HC (*n* = 15)**	**N-T1D (*n* = 7)**	**L-T1D (*n* = 9)**	**HT (*n* = 8)**	**GD (*n* = 7)**	**AD (*n* = 8)**
Age (yr)	28.9 ± 1.5	23.3 ± 1.0	34 ± 2.9	32 ± 2.9	34.4 ± 3.6	36.3 ± 2.5
Sex (M/F)	7/8	5/2	5/4	2/6	2/5	6/2
Disease duration (yr)	NA	0.3 ± 0.03	19.1 ± 2.4	4.2 ± 1.0	0.5 ± 0.13	6.3 ± 1.8
BMI (18.5–25 kg/m^2^)	22.9 ± 0.7	21.1 ± 0.6	26.2 ± 1.4	24.3 ± 1.1	26.7 ± 1.8	25.4 ± 1.6
Creatinine (60–105 μmol/L)	74.8 ± 3.4	65.0 ± 5.2	70.5 ± 3.2	67.0 ± 2.0	65.4 ± 4.7	85.5 ± 4.1
F-Glucose (4.0–6.0 mmol/L)	5.6 ± 0.1	11.3 ± 2.0	11.2 ± 1.0	5.9 ± 0.2	5.4 ± 0.3	5.2 ± 0.2
HbA1c (27–42 mmol/mol)	31.7 ± 0.6	75.4 ± 11.0	59.2 ± 3.0	32.5 ± 1.0	30.9 ± 1.4	32.9 ± 0.9
TSH (0.4–4.0 mIE/L)	1.9 ± 0.2	3.8 ± 1.0	2.3 ± 0.2	5.9 ± 2.4	1.0 ± 0.8	3.0 ± 0.4
T3 (3.1–6.8 pmol/L)	5.1 ± 0.1	5.4 ± 0.1	4.8 ± 0.2	4.5 ± 0.2	5.8 ± 1.2	6.3 ± 0.3
T4 (12.0–22.0 pmol/L)	15.6 ± 0.4	15.2 ± 0.8	15.0 ± 0.4	16.0 ± 0.9	20.4 ± 2.4	16.3 ± 1.0
S-Cortisol (220–650 nmol/L)	541 ± 71	497 ± 136	466 ± 25	425 ± 27	341 ± 27	526 ± 64

*Age, sex distribution, disease duration, BMI and clinical parameters at the time of sampling were assessed in healthy controls (HC) and patients with new-onset (N-T1D) and long-standing (L-T1D) type 1 diabetes, Hashimoto's thyroiditis (HT), Graves' disease (GD), and autoimmune Addison's disease (AD). Normal ranges for clinical parameters are described (first column). All values are given as mean ± SEM. F, female; HbA1c, glycated hemoglobin; M, male; TSH, thyroid-stimulating hormone; T3, free triiodothyronine; T4, free thyroxine; yr, years*.

### Blood Sampling and Preparation of PBMC

Peripheral venous blood was collected in three Vacuette® sodium-heparin tubes à 9 mL (Greiner Bio-One; Austria) in the morning (8–10 a.m.). All study subjects had been fasting since 10 p.m. the night before sampling. Blood samples were sent to the Pediatric research laboratory, Linköping University, at room-temperature and were processed within 27 h after sample collection. PBMC were obtained by performing density-gradient centrifugation with Leucosep® tubes (Greiner Bio One; Austria) and Ficoll-Paque™ PLUS (density 1.077 ± 0.001 g/mL, GE Healthcare; UK) according to manufacturer's instructions. Cells were washed in RPMI 1640 w/o L-glutamine (Gibco; US) supplemented with 2% (v/v) heat-inactivated FCS (Gibco; US). PBMC were incubated over-night in complete medium (RPMI 1640 w/o L-glutamine supplemented with 10% (v/v) heat-inactivated FCS) at 2 × 10^6^ cells/mL.

### Mass Cytometry Staining

The antibody panel, stimulation conditions, and intracellular staining used for mass cytometry in this study were optimized and validated as described elsewhere (18). Monoclonal antibodies and reagents are described in detail in Supplementary Table 1. Purified carrier-free antibodies were conjugated by using Maxpar® antibody labeling kit (Fluidigm; US) according to the manufacturer's instructions.

PBMC (1 × 10^7^) were pre-incubated with anti-CD4-^144^Nd for 30 min at room temperature to improve the anti-CD4 staining upon stimulation. Cells (5 × 10^6^ cells/condition) were left untreated or stimulated with 100 ng/mL PMA (Sigma-Aldrich; US) and 1 μg/mL ionomycin (Sigma-Aldrich; US) for 4 h at 37°C in 5% CO_2_ in the presence of 2 μM Monensin (Affymetrix eBioscience; US) and 3 μg/mL brefeldin A (Affymetrix eBioscience; US). PBMC were washed twice in PBS and stained with 2.5 μM Cell-ID™ Cisplatin (Fluidigm; US) live/dead discriminator for 5 min at room-temperature. The live/dead-staining was quenched by adding complete medium. Cells were then washed in Maxpar® Cell staining buffer (CSB, Fluidigm; US) and incubated in an extracellular staining cocktail (Supplementary Table 1) for 30 min at 4°C. PBMC were washed in CSB and resuspended in FOXP3 Fixation/Permeabilization buffer (Affymetrix eBioscience; US) for 40 min. Cells were then washed with Permeabilization buffer (Affymetrix eBioscience; US) and stained with an intracellular staining cocktail for 30 min at 4°C. Samples were washed twice in Permeabilization buffer, once in CSB and then fixed over-night in PBS with 2% paraformaldehyde (Novakemi AB; Sweden) at 4°C.

### Sample Barcoding and Data Acquisition

Barcoded sample batches were processed simultaneously to reduce data collection variability. Sample barcoding and data acquisition were carried out as previously described (18). Briefly, cells were washed twice in Maxpar® Barcode perm buffer (Fluidigm; US) and each sample was stained with a different barcode combination using Cell-ID 20-plex Pd-barcoding kit (Fluidigm; US) for 30 min at room temperature. After washing twice in CSB, samples were pooled together into one tube. Cells were incubated for 20 min at room temperature with 125 nM Cell-ID™ DNA Intercalator-Ir191/193 (Fluidigm; US), washed twice with CSB and once with Di water and lastly resuspended with 0.1 % EQ four element calibration beads (Fluidigm; US). Data were acquired with a CyTOF2® instrument (Fluidigm; US). After acquisition, data were concatenated, normalized using mass bead signal and de-barcoded using the CyTOF 2 software.

### Data Analysis and Statistics

Data of single, live CD45^+^ cells from each sample were manually gated using Cytobank (76) (Supplementary Figure 1A). Data were first hyperbolic ArcSinh-transformed with a cofactor of 5 and then a 5-level HSNE analysis was performed in Cytosplore^+HSNE^ (77) with 30 perplexity and 1,000 iterations (default settings). All markers were selected in HSNE analysis except cytokines (IFN-γ, IL-2, IL-4, IL-10, and IL-17A). Major cell lineages were identified at the overview level and analyzed separately in a data-driven manner (77). To facilitate subset identification and reduce computing time, CD4^+^ and CD8^+^ T cells were further divided into naïve and memory cells in subsequent levels according to the expression of CD45RA, CD45RO, CCR7, and CD27. Clustering was performed by GMS in Cytosplore. Clusters showing similar phenotype were manually merged. Further exploration of cluster frequencies and median ArcSinh5-transformed marker expression was performed using Cytofast (78). For cytokine expression, differential median ArcSinh5-expression between clusters with similar phenotype in unstimulated and stimulated samples was calculated. Shapio Wilk's normality test was used to evaluate data distribution. Chi-square test was used to assess differences in sex distribution between the study groups. One-way ANOVA and Tukey's test for multiple comparisons were applied for statistical analysis of data (α = 0.05). All statistical analyses were performed in GraphPad Prism 8 software.

### Study Approval

This study was approved by the Regional Research Ethical Committee in Uppsala (Dnr 2014/485) and was consistent with The Declaration of Helsinki. All participants gave their written informed consent prior to inclusion in the study.

## Data Availability Statement

The datasets generated for this study are available on request to the corresponding author.

## Ethics Statement

The studies involving human participants were reviewed and approved by Regional Research Ethical Committee in Uppsala. The patients/participants provided their written informed consent to participate in this study.

## Author Contributions

P-OC and DE were responsible for study conception and handling of clinical data. DE, P-OC, and SB recruited study subjects. LM, HB, and RC designed the immunological experiments. HB, RC, and MP designed the panel and protocol for mass cytometry. LM, HB, and MP performed mass cytometry experiments. Acquisition and analysis of data were performed by MP and HB, respectively. Data were interpreted by LM, HB, and RC. LM, RC, and HB wrote the manuscript. The manuscript was revised by DE and P-OC. Final approval for submission was given by all authors.

### Conflict of Interest

The authors declare that the research was conducted in the absence of any commercial or financial relationships that could be construed as a potential conflict of interest.
