# Supplementary material for: Mass Cytometry Studies of Patients With Autoimmune Endocrine Diseases Reveal Distinct Disease-Specific Alterations in Immune Cell Subsets
Source: Front Immunol. 2020 Feb 21;11:288. doi: 10.3389/fimmu.2020.00288 (PMC7047233; doi:10.3389/fimmu.2020.00288)
Supplement: Supplementary file 1 [file Presentation_1.pptx]

## Slide 1
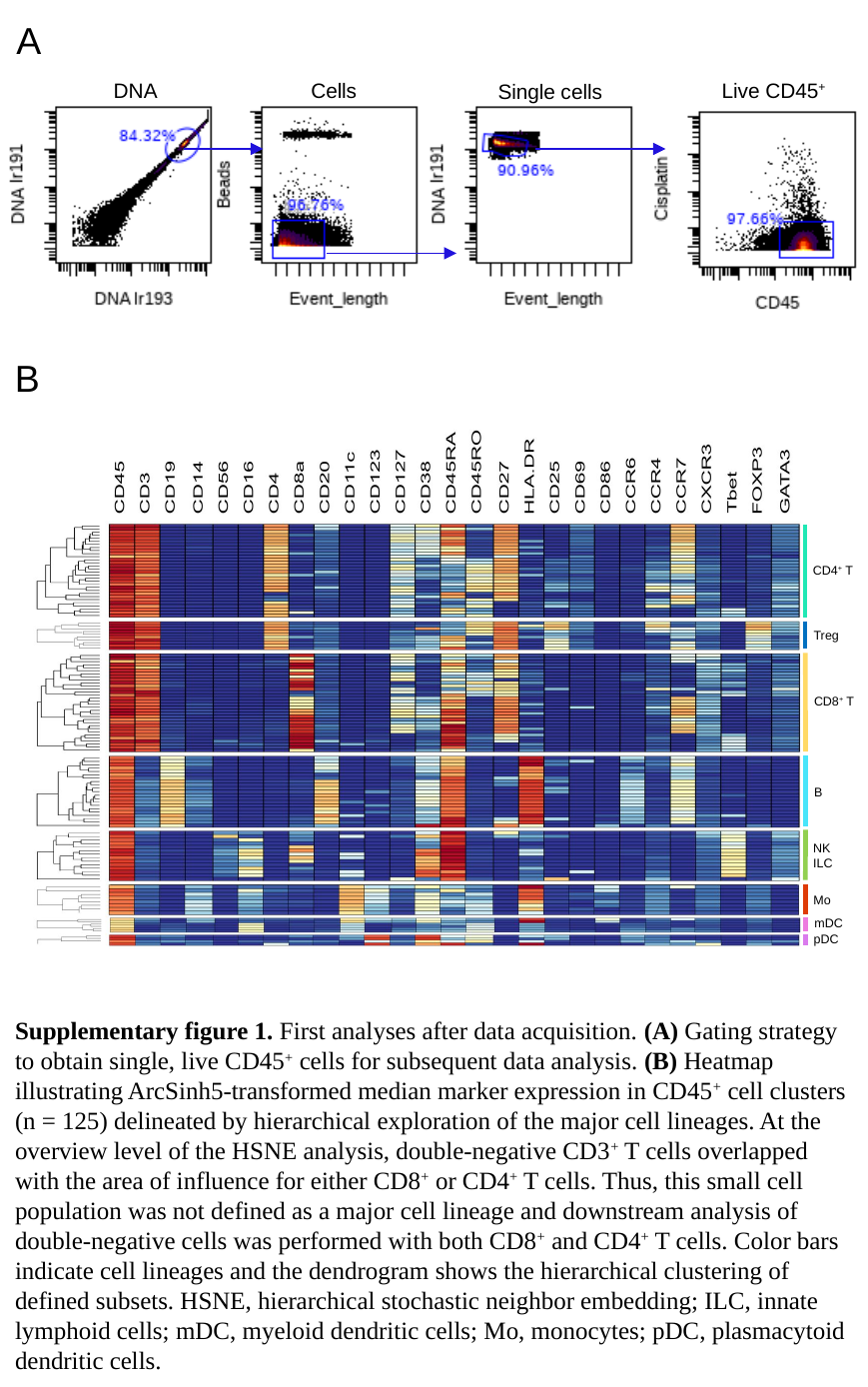

A
Live CD45+
DNA
Cells
Single cells
B
CD4+ T
Treg
CD8+ T
B
NK
ILC
Mo
mDC
pDC
Supplementary figure 1. First analyses after data acquisition. (A) Gating strategy to obtain single, live CD45+ cells for subsequent data analysis. (B) Heatmap illustrating ArcSinh5-transformed median marker expression in CD45+ cell clusters (n = 125) delineated by hierarchical exploration of the major cell lineages. At the overview level of the HSNE analysis, double-negative CD3+ T cells overlapped with the area of influence for either CD8+ or CD4+ T cells. Thus, this small cell population was not defined as a major cell lineage and downstream analysis of double-negative cells was performed with both CD8+ and CD4+ T cells. Color bars indicate cell lineages and the dendrogram shows the hierarchical clustering of defined subsets. HSNE, hierarchical stochastic neighbor embedding; ILC, innate lymphoid cells; mDC, myeloid dendritic cells; Mo, monocytes; pDC, plasmacytoid dendritic cells.

## Slide 2
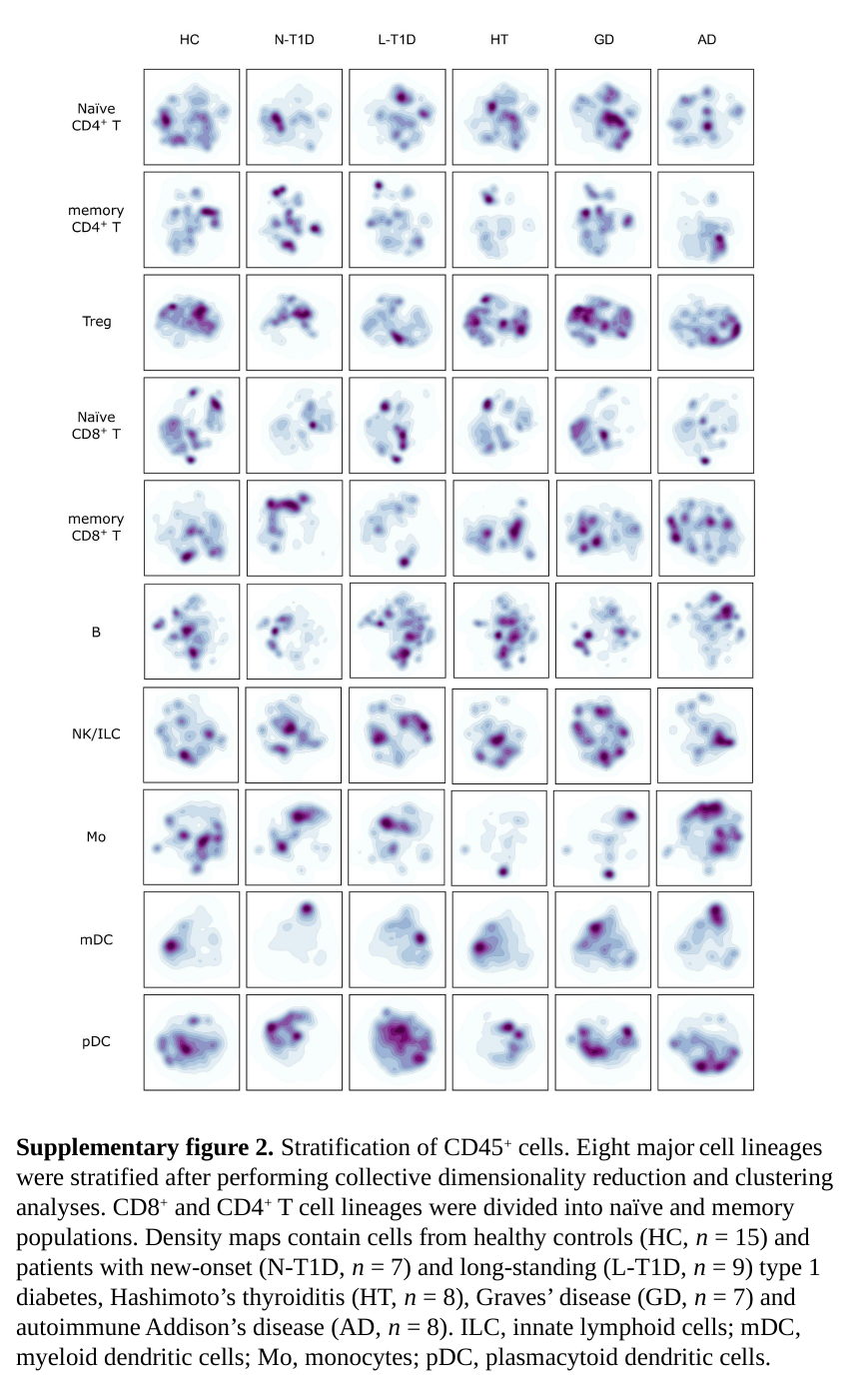

Supplementary figure 2. Stratification of CD45+ cells. Eight major cell lineages were stratified after performing collective dimensionality reduction and clustering analyses. CD8+ and CD4+ T cell lineages were divided into naïve and memory populations. Density maps contain cells from healthy controls (HC, n = 15) and patients with new-onset (N-T1D, n = 7) and long-standing (L-T1D, n = 9) type 1 diabetes, Hashimoto’s thyroiditis (HT, n = 8), Graves’ disease (GD, n = 7) and autoimmune Addison’s disease (AD, n = 8). ILC, innate lymphoid cells; mDC, myeloid dendritic cells; Mo, monocytes; pDC, plasmacytoid dendritic cells.

## Slide 3
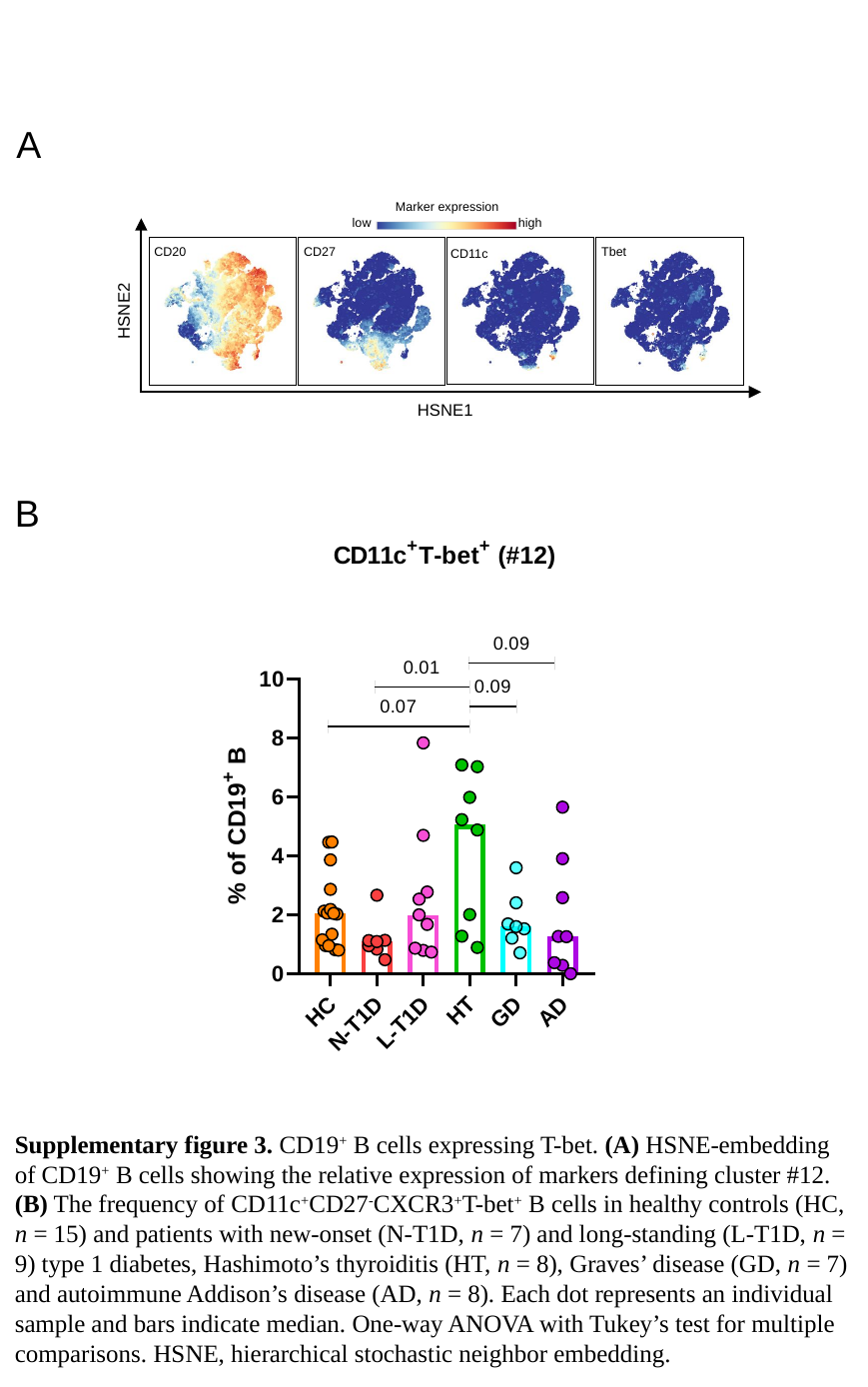

A
Marker expression
high
low
Tbet
CD20
CD27
CD11c
HSNE2
HSNE1
B
Supplementary figure 3. CD19+ B cells expressing T-bet. (A) HSNE-embedding of CD19+ B cells showing the relative expression of markers defining cluster #12. (B) The frequency of CD11c+CD27-CXCR3+T-bet+ B cells in healthy controls (HC, n = 15) and patients with new-onset (N-T1D, n = 7) and long-standing (L-T1D, n = 9) type 1 diabetes, Hashimoto’s thyroiditis (HT, n = 8), Graves’ disease (GD, n = 7) and autoimmune Addison’s disease (AD, n = 8). Each dot represents an individual sample and bars indicate median. One-way ANOVA with Tukey’s test for multiple comparisons. HSNE, hierarchical stochastic neighbor embedding.

## Slide 4
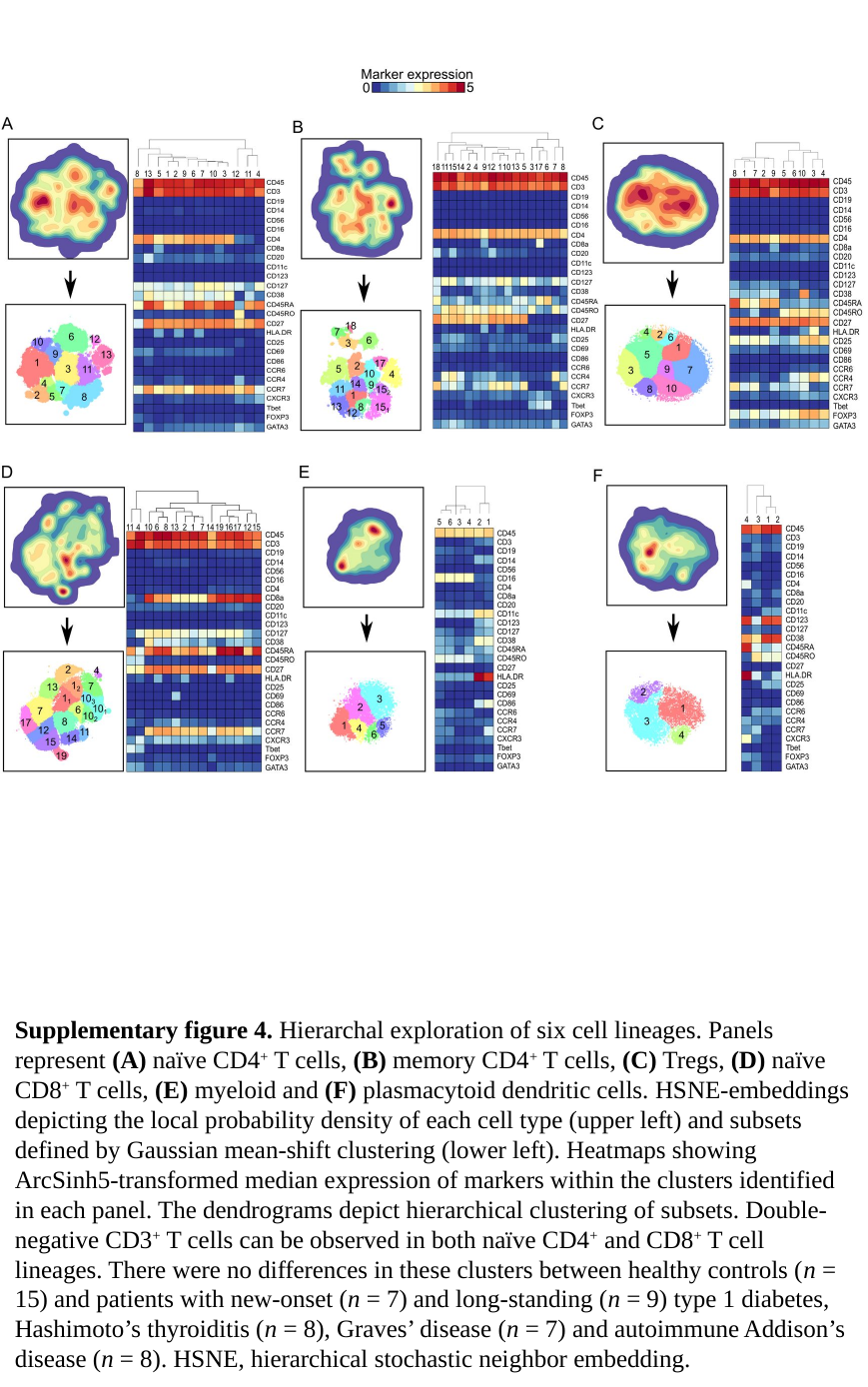

Supplementary figure 4. Hierarchal exploration of six cell lineages. Panels represent (A) naïve CD4+ T cells, (B) memory CD4+ T cells, (C) Tregs, (D) naïve CD8+ T cells, (E) myeloid and (F) plasmacytoid dendritic cells. HSNE-embeddings depicting the local probability density of each cell type (upper left) and subsets defined by Gaussian mean-shift clustering (lower left). Heatmaps showing ArcSinh5-transformed median expression of markers within the clusters identified in each panel. The dendrograms depict hierarchical clustering of subsets. Double-negative CD3+ T cells can be observed in both naïve CD4+ and CD8+ T cell lineages. There were no differences in these clusters between healthy controls (n = 15) and patients with new-onset (n = 7) and long-standing (n = 9) type 1 diabetes, Hashimoto’s thyroiditis (n = 8), Graves’ disease (n = 7) and autoimmune Addison’s disease (n = 8). HSNE, hierarchical stochastic neighbor embedding.

## Slide 5
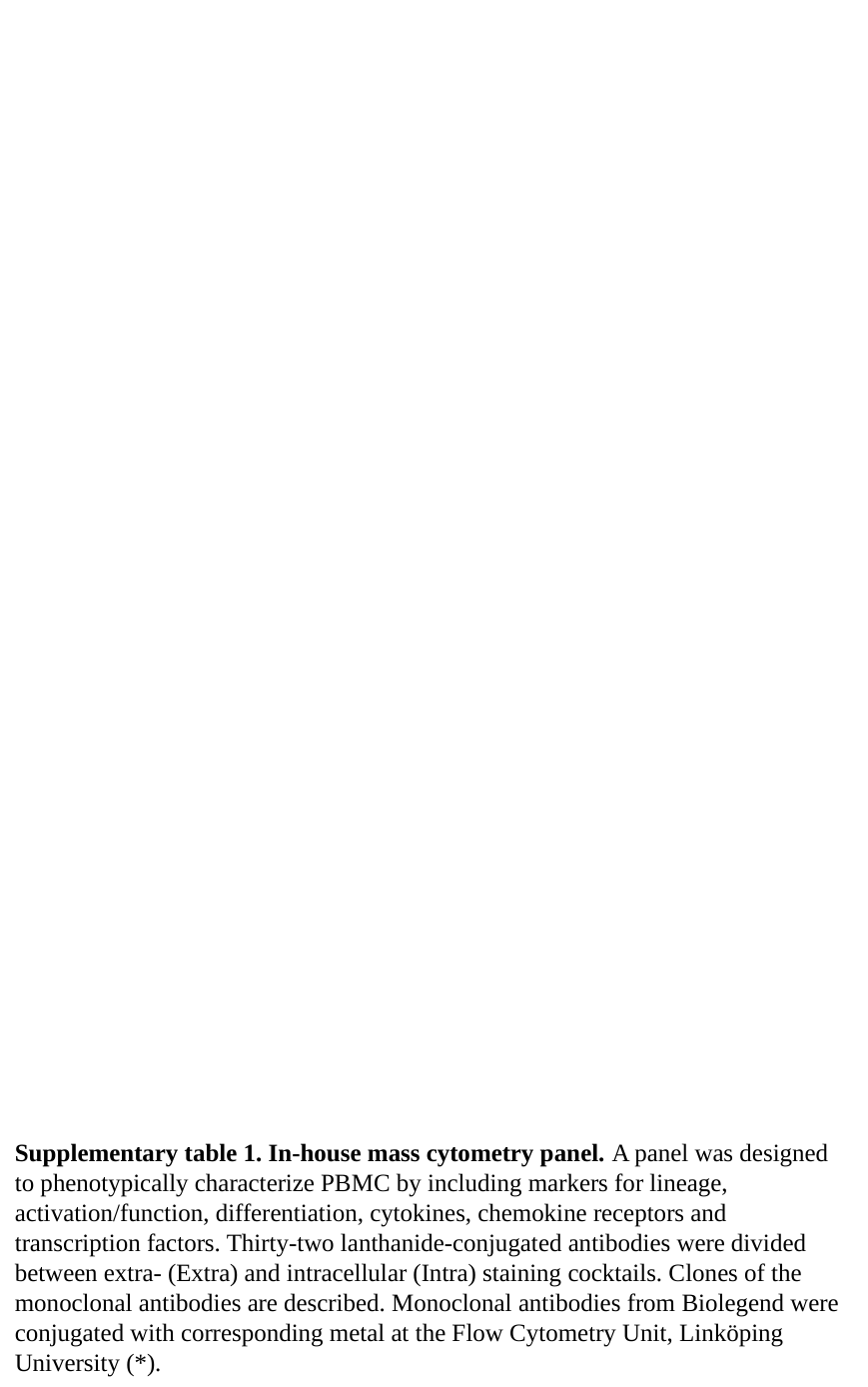

Supplementary table 1. In-house mass cytometry panel. A panel was designed to phenotypically characterize PBMC by including markers for lineage, activation/function, differentiation, cytokines, chemokine receptors and transcription factors. Thirty-two lanthanide-conjugated antibodies were divided between extra- (Extra) and intracellular (Intra) staining cocktails. Clones of the monoclonal antibodies are described. Monoclonal antibodies from Biolegend were conjugated with corresponding metal at the Flow Cytometry Unit, Linköping University (*).

## Slide 6
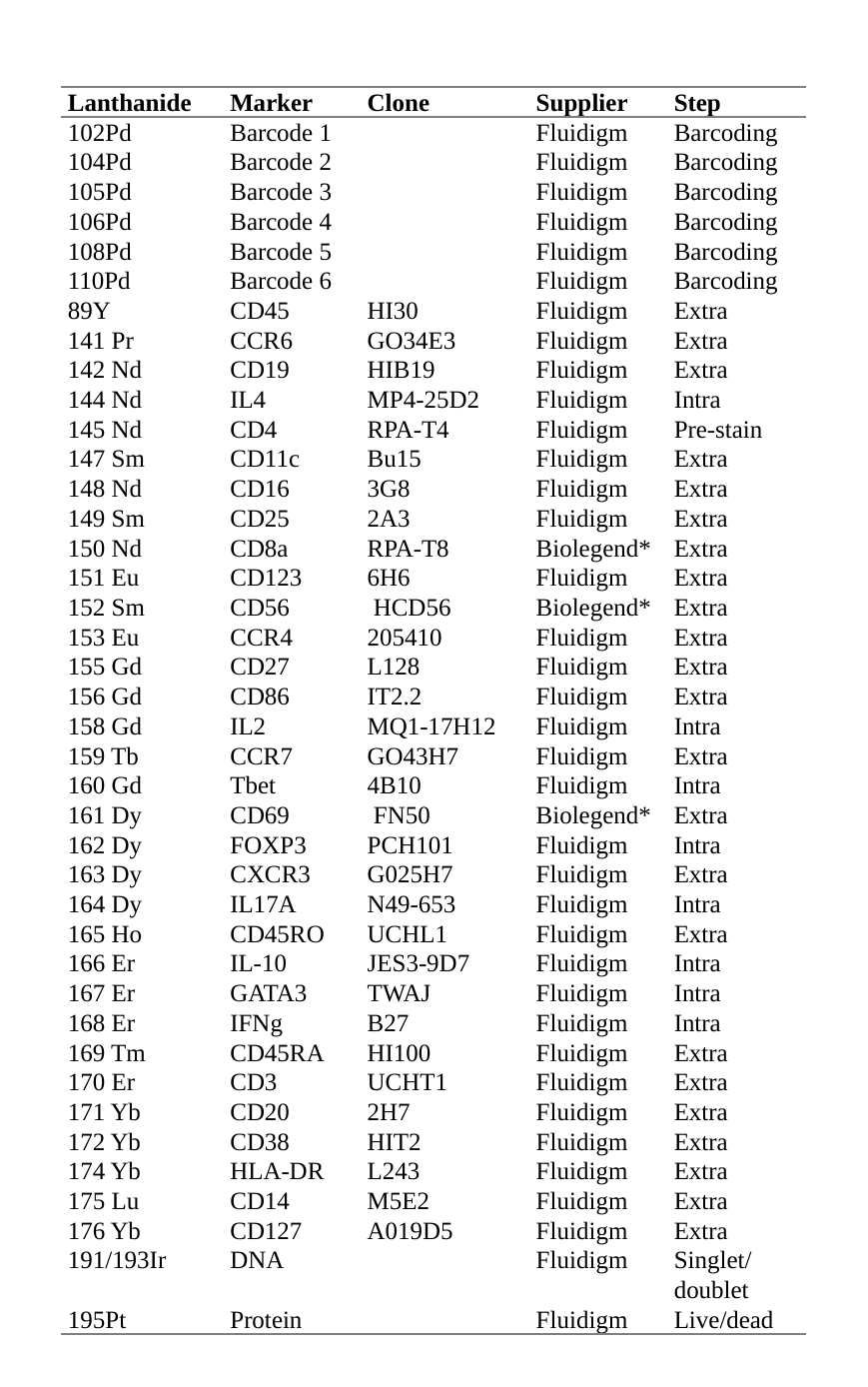

| Lanthanide | Marker | Clone | Supplier | Step |
| --- | --- | --- | --- | --- |
| 102Pd | Barcode 1 | | Fluidigm | Barcoding |
| 104Pd | Barcode 2 | | Fluidigm | Barcoding |
| 105Pd | Barcode 3 | | Fluidigm | Barcoding |
| 106Pd | Barcode 4 | | Fluidigm | Barcoding |
| 108Pd | Barcode 5 | | Fluidigm | Barcoding |
| 110Pd | Barcode 6 | | Fluidigm | Barcoding |
| 89Y | CD45 | HI30 | Fluidigm | Extra |
| 141 Pr | CCR6 | GO34E3 | Fluidigm | Extra |
| 142 Nd | CD19 | HIB19 | Fluidigm | Extra |
| 144 Nd | IL4 | MP4-25D2 | Fluidigm | Intra |
| 145 Nd | CD4 | RPA-T4 | Fluidigm | Pre-stain |
| 147 Sm | CD11c | Bu15 | Fluidigm | Extra |
| 148 Nd | CD16 | 3G8 | Fluidigm | Extra |
| 149 Sm | CD25 | 2A3 | Fluidigm | Extra |
| 150 Nd | CD8a | RPA-T8 | Biolegend\* | Extra |
| 151 Eu | CD123 | 6H6 | Fluidigm | Extra |
| 152 Sm | CD56 | HCD56 | Biolegend\* | Extra |
| 153 Eu | CCR4 | 205410 | Fluidigm | Extra |
| 155 Gd | CD27 | L128 | Fluidigm | Extra |
| 156 Gd | CD86 | IT2.2 | Fluidigm | Extra |
| 158 Gd | IL2 | MQ1-17H12 | Fluidigm | Intra |
| 159 Tb | CCR7 | GO43H7 | Fluidigm | Extra |
| 160 Gd | Tbet | 4B10 | Fluidigm | Intra |
| 161 Dy | CD69 | FN50 | Biolegend\* | Extra |
| 162 Dy | FOXP3 | PCH101 | Fluidigm | Intra |
| 163 Dy | CXCR3 | G025H7 | Fluidigm | Extra |
| 164 Dy | IL17A | N49-653 | Fluidigm | Intra |
| 165 Ho | CD45RO | UCHL1 | Fluidigm | Extra |
| 166 Er | IL-10 | JES3-9D7 | Fluidigm | Intra |
| 167 Er | GATA3 | TWAJ | Fluidigm | Intra |
| 168 Er | IFNg | B27 | Fluidigm | Intra |
| 169 Tm | CD45RA | HI100 | Fluidigm | Extra |
| 170 Er | CD3 | UCHT1 | Fluidigm | Extra |
| 171 Yb | CD20 | 2H7 | Fluidigm | Extra |
| 172 Yb | CD38 | HIT2 | Fluidigm | Extra |
| 174 Yb | HLA-DR | L243 | Fluidigm | Extra |
| 175 Lu | CD14 | M5E2 | Fluidigm | Extra |
| 176 Yb | CD127 | A019D5 | Fluidigm | Extra |
| 191/193Ir | DNA | | Fluidigm | Singlet/ doublet |
| 195Pt | Protein | | Fluidigm | Live/dead |
